# What’s Weight Got to Do With It? Mental Health Trainees’ Perceptions of a Client With Anorexia Nervosa Symptoms

**DOI:** 10.3389/fpsyg.2018.02574

**Published:** 2018-12-17

**Authors:** Laurie A. S. Veillette, Jose Martinez Serrano, Paula M. Brochu

**Affiliations:** College of Psychology, Nova Southeastern University, Fort Lauderdale, FL, United States

**Keywords:** weight stigma, eating disorders, anorexia nervosa, mental healthcare, weight stereotypes

## Abstract

This study examined the effect of client body mass index (BMI) on diagnostic impressions and perceptions of mental health trainees. Participants read a vignette of a mock female client presenting for treatment with symptoms of anorexia nervosa. Participants were randomly assigned to one of three conditions in which the client was described as “underweight,” “normal weight,” or “overweight.” Results revealed that participants assigned to the “underweight” condition diagnosed the client with anorexia nervosa or atypical anorexia nervosa more frequently than participants assigned to the “overweight” or “normal weight” conditions. There was no difference based on client BMI when the more general diagnosis of other specified feeding or eating disorder (OSFED; previously known as eating disorder not otherwise specified [EDNOS]) was included, however. Participants in the “overweight” and “normal weight” conditions recommended fewer therapy sessions for the client than participants in the “underweight” condition. Furthermore, participants more strongly endorsed weight-based stereotypes to describe the client when she was “overweight” than “normal weight” or “underweight.” Contrary to hypotheses, however, participants reported moderately positive attitudes toward treating the client regardless of BMI. These preliminary findings support initiatives aimed at providing training on weight stigma and eating disorders to mental health professionals.

## Introduction

Weight stigma, which includes negative attitudes, beliefs, and behaviors toward people perceived to carry excess weight, is widely accepted and prevalent in society (Puhl and Heuer, 2009). Although weight stigma can be experienced and internalized by anyone of any size, it is greater among women and increases as body mass index (BMI) increases (Puhl et al., 2008). Weight stigma is prevalent in both medical and mental health settings, influencing professionals’ attitudes toward higher body-weight clients and clients’ views of treatment, potentially impairing the quality and frequency of care (Puhl and Heuer, 2010; Phelan et al., 2015). Stigma associated with higher body-weight mirrors the stigma that is associated with people with disordered eating behaviors, as both are perceived as imperfections of personal character and distortions of the body (Goffman, 1963; Puhl and Suh, 2015). Disordered eating behaviors among people of a higher body-weight, including severe dietary restriction, are often unrecognized, misdiagnosed, or otherwise overlooked by healthcare professionals (Sim et al., 2013; Lebow et al., 2015). Given the scarcity of research examining weight stigma in the mental health field and, in particular, the conceptualization and treatment of restrictive eating disorders like anorexia nervosa, this study was conducted to shed light on these issues.

Weight stigma is associated with a number of physical and psychological health consequences (Puhl and Heuer, 2010; Hunger et al., 2015). It is associated with low self-esteem, body image dissatisfaction, lower quality of life, and eating, weight, and shape concerns (Puhl and Heuer, 2009; Roberto et al., 2012). Weight stigma increases the risk for mood disorders, anxiety disorders, and substance use disorders, with nearly one-third of higher-body weight participants who perceived weight discrimination in the past year meeting criteria for these psychological disorders (Hatzenbuehler et al., 2009). Weight stigma mediates the association between BMI and physical health and psychological well-being (Hunger and Major, 2015), and perceived weight discrimination has even been linked to shortened life expectancy independent of BMI (Sutin et al., 2015).

Weight stigma is pervasive in health care settings and has a profound impact on client care. For example, physicians report negative perceptions of higher body-weight clients being “weak-willed, sloppy, or lazy” as well as “awkward, unattractive, ugly, and non-compliant,” along with relatively poor expectations for treatment outcomes (Foster et al., 2003, p. 1173). Other studies have found that healthcare providers spend less time educating higher body-weight clients, report having less respect for them, and prescribe different treatment recommendations compared to lower body-weight clients (Phelan et al., 2015). In addition to weight stigma, these discrepancies in medical care and client-doctor interactions are likely perpetuated by weight-based health models which posit that body size equates to general physical health (Bacon and Aphramor, 2011; Lee and Pausé, 2016). This perspective fails to consider the multidimensionality of health and well-being and predisposes medical providers to erroneously over-attribute health issues to weight.

Although few studies have examined weight stigma among mental health professionals, available research generally shows that clinical and counseling psychologists report lower expectations for higher body-weight clients’ prognosis, effort, and functioning, and assign more negative psychological symptoms and diagnostic judgments to them (Brochu et al., 2018). For example, Agell and Rothblum (1991) found that psychologists perceive higher body-weight clients as more embarrassed, unattractive, and kind than lower body-weight clients, reflecting common weight stereotypes. Like other health professionals, eating disorder specialists endorse negative weight stereotypes (e.g., overeat, insecure, inactive, poor self-control) and report lower treatment expectations for higher body-weight clients (Puhl et al., 2013a). Considering that higher body-weight and weight stigma are both reliable risk factors for eating disorder symptomatology, weight stigma within psychological health contexts is particularly concerning (Haines and Neumark-Sztainer, 2006; Alberga et al., 2016).

Counter to common perceptions about eating disorders, several studies have found that people of a higher body-weight are *more* susceptible to developing eating disorders than all other weight groups (Neumark-Sztainer et al., 2006; Darby et al., 2007; Sim et al., 2013; Lebow et al., 2015). For example, a recent study by Lipson and Sonneville (2017) found that among 9,713 students across 12 US colleges and universities, body weight was the most reliable predictor of eating disorder risk, with higher body-weight students reporting more disordered eating symptoms. Despite these findings, weight bias remains prevalent in the treatment of eating disorders for people of a higher body-weight, as healthcare providers continue to overlook, misdiagnose, and even encourage disordered eating symptoms for their higher body-weight clients (Burgard, 2004; Sim et al., 2013). In part, this is likely due to the diagnostic requirements of the Diagnostic and Statistical Manual of Mental Disorders, Fifth Edition (DSM-5) set forth by the American Psychiatric Association [APA] (2013, p. 338), such as the criterion that a person be of a “significantly low body weight” to warrant an anorexia nervosa diagnosis. Those who do not meet this criterion are likely to be diagnosed with atypical anorexia nervosa under the other specified feeing or eating disorder (OSFED; previously known as eating disorder not otherwise specified [EDNOS]) classification, which is often perceived as less severe and, thus, can impede access to immediate and appropriate eating disorder treatment (Fairburn and Bohn, 2005). Although it is well-known that anorexia nervosa is serious because of medical complications, research demonstrates that EDNOS has elevated mortality risks similar to those found in anorexia nervosa and bulimia nervosa (Crow et al., 2009), and that those who do not meet diagnostic criteria for anorexia nervosa because of higher-weight status exhibit similar profiles of life-threatening complications as those diagnosed with anorexia nervosa (Peebles et al., 2010; Whitelaw et al., 2014).

In considering the influence of weight stigma on eating disorders, it is important to be mindful that eating disorders in and of themselves are also stigmatizing. Similar to weight stigma, eating disorder stigma is widespread and is frequently perpetuated by stereotypes portrayed by the media (Puhl and Suh, 2015). These stereotyped representations often include a thin, Caucasian woman presenting with anorexia nervosa or bulimia nervosa symptoms (Gordon et al., 2002). Eating disorder stigma is reinforced by common misperceptions about the controllability of these disorders and is also influenced by the type and severity of the eating disorder (Fleming and Szmukler, 1992; Crisafulli et al., 2008; Wingfield et al., 2011; Griffiths et al., 2015). For example, in a study of 317 participants with self-reported eating disorders, researchers found a positive association between participants’ frequency of stigmatizing experiences related to their eating disorder and eating disorder severity, duration of eating disorder symptoms, and self-esteem (Griffiths et al., 2015). People with eating disorders share an increased risk of social support loss and difficulty accessing adequate medical and mental health treatment (Crisp et al., 2000; Mond et al., 2006). This is particularly the case for those who do not fit the traditional eating disorder stereotype (e.g., people of a “normal” or higher body-weight), whose symptoms are more likely to be dismissed by society and the health care community (Sim et al., 2013; Lebow et al., 2015).

The present study examines whether client BMI affects mental health trainees’ perceptions of a client presenting with anorexia nervosa symptoms. Given the prevalence of weight stigma and its presence in healthcare settings, this research is both relevant and necessary. Participants read a clinical vignette in which a hypothetical female client, presenting with symptoms of anorexia nervosa, was described as “underweight,” “normal weight,” or “overweight.” Consistent with research indicating that restrictive eating disorders are overlooked by healthcare providers when reported by people of a higher body-weight, it was hypothesized that participants would disproportionately alternatively diagnose symptoms of anorexia nervosa when the client was described as “overweight.” In consideration of the research literature showing that healthcare providers recommend fewer treatment sessions to clients of a higher body-weight, it was also hypothesized that mental health trainees would recommend fewer treatment sessions for the higher body-weight client. Given the pervasiveness of weight stigma in healthcare settings, it was hypothesized that participants would report more weight-based stereotypes for the higher body-weight client. Finally, it was hypothesized that participants would report having less positive attitudes toward working with the client when she was described as being of a higher body-weight. This is consistent with research demonstrating the negative perceptions and expectations reported by healthcare providers in working with people of a higher body-weight.

## Materials and Methods

### Participants

Participants were recruited from graduate-level mental health program social media sites (e.g., Facebook), email, and flyer dispersal at a university in the southeastern United States. Participants included 90 graduate students (81 women; *M* age = 26.15, *SD* = 3.65) recruited from mental health programs (77 in a doctoral clinical psychology program, three in a Master’s level mental health counseling program, two in a Master’s level counseling program, and one in a doctoral level school psychology program). The majority of the sample (*n* = 56; 62%) identified as white; of the remaining participants, 16 (18%) identified as Hispanic/Latinx, six (7%) identified as multiracial, five (6%) identified as African American, four (4%) identified as Asian, and three (3%) declined to report their race/ethnicity. Of the 90 participants, 16 (18%) reported being in the first year of their training program, 17 (19%) reported being in their second year, 16 (18%) reported being in their third year, 22 (24%) reported being in their fourth year, 14 (16%) reported being in their fifth year (typically internship), and one (1%) reported being in their sixth year. Four participants (4%) did not report their program year. Of the 90 participants, 25 (28%) indicated that their primary focus was in eating disorders, while other participants reported a wide array of interests in areas such as trauma, mood disorders, personality disorders, and working with children/adolescents.

### Measures

#### Diagnosis

Participants reported what diagnosis they would assign the client based on the vignette (Agell and Rothblum, 1991). The measure is comprised of one, open-ended question (i.e., *What would your diagnosis for Susan be?*).

#### Number of Treatment Sessions

Participants were asked to report how many treatment sessions they would recommend for the client ranging from 0 to 24 (Agell and Rothblum, 1991).

#### Weight Stereotyping

Participants were asked to complete a shortened version of the Fat Phobia Scale to report their perceptions of the client in the vignette (Bacon et al., 2001). Instead of being asked to indicate to what extent participants thought a series of adjectives described fat people, participants were asked to report to what extent each set of adjectives described the client (e.g., *willpower – no willpower, active – inactive, dislikes food – likes food*). Participants responded to each item on a semantic differential scale of 1 to 5. Higher scores indicate more application of weight stereotypes to the client (Cronbach’s α = 0.84).

#### Treatment Attitudes

Participants were asked to report their attitudes toward treating the client on 13 items modified from Puhl et al. (2013a) and Puhl et al. (2013b; e.g., *Treating Susan would be professionally rewarding*; see Supplementary Material for modified items). Participants responded to each item on a 7-point Likert scale of 1 (*strongly disagree*) to 7 (*strongly agree*). Higher scores on this measure indicate more positive attitudes toward treating the client. In the present study, this measure was internally reliable as indicated by a Cronbach’s α of 0.82.

### Procedure

Interested mental health graduate students clicked on the survey link on the recruitment flyer, directing them to the participation letter upon which they gave informed consent for voluntarily participating in the study. Informed consent was obtained electronically from participants in this study. Participants completed the study online and were randomly assigned to read one of three vignettes in which the client was described as having symptoms of anorexia nervosa. The vignettes were developed for the purpose of the study by attending to the diagnostic criteria for anorexia nervosa outlined in the DSM-5 (American Psychiatric Association [APA], 2013; see Supplementary Material for the vignette). For example, the client, Susan, presented with a restricted diet of no more than 800 calories per day for the previous six months, weighed herself multiple times per day, measured her waist, buttocks, arms, and legs weekly, and reported being terrified of gaining weight, stating that “she would rather die than get fat and ugly.” Each of the three vignettes maintained the same clinical presentation for the client but differed by her reported weight/BMI. Susan was described as “underweight” (BMI = 16.6, *n* = 31), “normal weight” (BMI = 21.3, *n* = 25), or “overweight” (BMI = 29.5, *n* = 34). According to diagnostic criteria outlined in the DSM-5 (American Psychiatric Association [APA], 2013), a diagnosis of anorexia nervosa or atypical anorexia nervosa would be considered an appropriate diagnosis, depending on the client’s weight, based on the information provided in the vignette. After reading the vignette, participants then completed a series of measures described below, a demographics questionnaire, and were debriefed. All study procedures were approved by the Institutional Review Board (IRB) of Nova Southeastern University.

## Results

### Preliminary Analyses

The measures were aggregated by first reverse-coding the necessary items and then total scores were created by calculating the mean of the items, following the instructions of the scale authors. Diagnoses ascribed to the client by participants were systematically coded by the researchers (i.e., 1 = *anorexia nervosa*, 2 = *atypical anorexia nervosa*, 3 = *avoidant/restrictive food intake disorder*, 4 = *eating disorder not otherwise/otherwise specified*, 5 = *body dysmorphic disorder*, 6 = *bulimia nervosa*, 7 = *adjustment disorder*, 8 = *obsessive compulsive disorder*, 9 = *no diagnosis provided*). Eighty-seven participants (97%) correctly identified the client’s BMI in a manipulation check, and 82 (91%) correctly identified details from the vignette (e.g., Susan’s marital status) in four comprehension checks, indicating that participants read the vignette and attended to its content. Excluding participants who failed the manipulation or comprehension checks does not modify the findings reported below.

### Diagnosis

It was hypothesized that mental health trainees would be more likely to alternatively diagnose or overlook symptoms of anorexia nervosa reported by a higher body-weight client. Participants assigned differing diagnoses to the client depending on her BMI, χ^2^(16, *N* = 90) = 34.11, *p* = 0.005 (see Table 1). To determine how the BMI conditions differed from each other, *post hoc* analyses were conducted following the recommendations of Beasley and Schumacker (1995) using adjusted standardized residuals converted to χ^2^ values. The planned comparison of assigning a diagnosis of anorexia nervosa or atypical anorexia nervosa as a function of BMI condition was of particular interest, so the *post hoc* analyses focused on these comparisons. Participants in the “overweight” condition were less likely to assign a diagnosis of anorexia nervosa or atypical anorexia nervosa, χ^2^(1) = 8.88, *p* = 0.003, whereas participants in the “underweight” condition were more likely to assign a diagnosis of anorexia nervosa or atypical anorexia nervosa, χ^2^(1) = 7.84, *p* = 0.005. Participants in the “normal weight” condition were no more nor less likely to assign a diagnosis of anorexia nervosa or atypical anorexia nervosa than another diagnosis, χ^2^(1) = 0.01, *p* = 0.904. Thus, the hypothesis that mental health trainees would be more likely to underestimate or overlook the symptoms of a higher body-weight client presenting with symptoms of anorexia nervosa was supported.

**Table 1:** Diagnosis as a function of client BMI.

Diagnosis	Underweight (*n* = 31)	Normal Weight (*n* = 25)	Overweight (*n* = 34)
Anorexia nervosa	24 (77%)	8 (32%)	10 (29%)
Atypical anorexia nervosa	0	7 (28%)	5 (15%)
Avoidant/restrictive food intake disorder	1 (3%)	1 (4%)	5 (15%)
Eating disorder not otherwise/other specified	2 (7%)	5 (20%)	10 (29%)
Body dysmorphic disorder	1 (3%)	2 (8%)	1 (3%)
Bulimia nervosa	0	1 (4%)	0
Adjustment disorder	0	0	1 (3%)
Obsessive compulsive disorder	0	0	1 (3%)
No diagnosis provided	3 (10%)	1 (4%)	1 (3%)


Some participants assigned a general diagnosis of OSFED or EDNOS to the client. As atypical anorexia nervosa falls under this umbrella category, a more conservative test of our hypothesis would examine the assignment of a diagnosis of anorexia nervosa, atypical anorexia nervosa, or OSFED/EDNOS as a function of BMI condition. Participants were as likely to assign one of these diagnoses to the client regardless of her BMI, χ^2^(2, *N* = 90) = 1.07, *p* = 0.587. The majority of participants (*n* = 71, 79%) assigned one of these three diagnoses to the client.

### Number of Treatment Sessions

It was hypothesized that mental health trainees would recommend fewer treatment sessions when the client was described as having a higher body-weight. To examine whether the number of treatment sessions recommended varied based upon client BMI, a one-way ANOVA was conducted, revealing a significant effect, *F*(2,86) = 3.39, *p* = 0.038, η^2^ = 0.07. Participants recommended fewer treatment sessions for the client when she was “overweight” (*M* = 15.32, *SD* = 4.20) than when she was “underweight” (*M* = 18.83, *SD* = 5.88), *t*(62) = 2.53, *p* = .013, *d* = 0.69. Participants recommended fewer treatment sessions for the client when she was “normal weight” (*M* = 16.16, *SD* = 6.63) than “underweight,” *t*(53) = 1.78, *p* = 0.078, *d* = 0.43, although this finding was not statistically significant. There was no difference in the number of sessions recommended when the client was “normal weight” or “overweight,” *t*(57) = 0.57, *p* = 0.568, *d* = 0.15. Thus, the hypothesis that mental health trainees would recommend fewer treatment sessions for a higher body-weight client presenting with symptoms of anorexia nervosa was supported.

### Weight Stereotyping

It was hypothesized that mental health trainees would report more weight-based stereotypical perceptions of the client when she was described as having a higher body-weight. To examine whether weight-based stereotypical perceptions of the client varied based upon client BMI, a one-way ANOVA was conducted, revealing a significant effect, *F*(2,87) = 5.16, *p* = 0.008, η^2^ = 0.11. Participants more strongly endorsed weight stereotypes in their perceptions of the client when she was “overweight” (*M* = 2.65, *SD* = 0.30) than when she was “normal weight” (*M* = 2.46, *SD* = 0.30) or “underweight” (*M* = 2.42, *SD* = 0.32), both *t*s > 2.37, *p*s < 0.020, *d*s > 0.63. There was no difference in weight stereotyping when the client was described as “normal weight” or “underweight,” *t*(54) = 0.44, *p* = 0.659, *d* = 0.13. Thus, the hypothesis that mental health trainees would report more weight-based stereotypical perceptions of a higher body-weight client presenting with symptoms of anorexia nervosa was supported.

### Treatment Attitudes

It was hypothesized that mental health trainees would report less positive attitudes toward treating the client presented in the vignette when she was described as having a higher body-weight. To examine whether participant attitudes varied based upon client BMI, a one-way ANOVA was conducted, revealing a non-significant difference, *F*(2,87) = 1.63, *p* = 0.202, η^2^ = 0.04. Participants reported similarly positive attitudes toward the client regardless of BMI (“underweight” *M* = 4.90, *SD* = 0.88; “normal weight” *M* = 5.22, *SD* = 1.02; “overweight” *M* = 5.27, *SD* = 0.75). Overall, treatment attitudes were moderately positive. Thus, the hypothesis that mental health trainees would report less positive attitudes toward treating a higher-body weight client presenting with symptoms of anorexia nervosa was not supported.

## Discussion

The present research study lends support to extant literature demonstrating weight stigma in clinical care, treatment, and the perceptions of healthcare providers (Brochu et al., 2018). This study is one of the first of its kind to demonstrate this effect in the conceptualization and treatment of restrictive eating disorders. Among mental health trainees, client body weight did, in fact, impact diagnosis, treatment recommendations, and stereotypical perceptions of a client presenting with anorexia nervosa symptoms. Participants were less likely to assign a diagnosis of anorexia nervosa or atypical anorexia nervosa to the higher-weight client. Participants’ assignment of diagnosis to the client did not vary by BMI condition when the general diagnosis of OSFED or EDNOS was classified as an “accurate” diagnosis. It is unclear what specific eating disorder participants were conceptualizing when they assigned this general diagnosis. It may be that participants were thinking about a diagnosis of atypical anorexia nervosa but did not specify. It is also possible that participants were considering other OSFED diagnoses, such as bulimia nervosa or binge eating disorder (of low frequency and/or limited duration). Furthermore, participants recommended fewer treatment sessions and assigned more weight-based stereotypical traits to the higher-weight client. However, contrary to hypotheses, participants’ attitudes were moderately positive toward the client regardless of BMI.

The present study sheds light on the impact of weight stigma on eating disorder treatment – namely how weight stigma can serve as a barrier to appropriate clinical care for a serious mental health disorder. This is demonstrated by participants’ ascriptions of differential diagnoses and recommendations of fewer treatment sessions for higher-weight clients presenting with anorexia nervosa symptoms. Given the diagnostic requirements for anorexia nervosa as determined by the DSM-5 (i.e., that a person have “significantly low weight;” American Psychiatric Association [APA], 2013, p. 338), it may not be surprising that so few participants in the “overweight” condition diagnosed the client as having anorexia nervosa. However, this did not lead participants in the higher body-weight condition to assign a diagnosis of atypical anorexia nervosa, which suggests that people may not readily consider atypical clinical presentations. This finding demonstrates how the strong emphasis placed on weight for eating disorder diagnosis can translate to a misunderstanding or overlooking of symptoms for people who are not considered to have a significantly low weight. As a result, symptoms may be prolonged, resulting in serious medical complications, and even possibly death (Lebow et al., 2015; Whitelaw et al., 2014). This is supported by studies that have found that people of various weight groups presenting with symptoms of anorexia nervosa share comparable weight loss-related medical problems (e.g., poor cardiovascular, neurological, and pulmonary functioning; refeeding syndrome), requirements of hospitalization, and mortality rates (Crow et al., 2009; Peebles et al., 2010; Whitelaw et al., 2014; Lebow et al., 2015).

The finding that participants were more likely to endorse stereotypical perceptions of a higher body-weight client has considerable implications for the mental health field. Not only do these perceptions likely influence clinical understanding, treatment planning, and general client care, there is evidence that provider perceptions are often obvious to clients (Phelan et al., 2015; Ali et al., 2016). Therefore, it should be considered that weight-based stereotypes maintained by mental health providers may be salient to clients, as well. This finding is particularly concerning as weight stigma from providers may compound internalized weight bias and associated feelings of shame, and may even deter people of a higher body-weight from engaging in health-promoting behaviors, such as seeking out mental health treatment.

Although this study utilized an experimental design and recruited a sample of mental health trainees, several limitations may limit the generalizability of results. The use of one vignette allowed for consistency between conditions, but future research may test a more diverse set of anorexia nervosa symptom presentations, as well as perceptions of other eating disorders. The vignette provided participants with limited information that did not provide as much understanding of the client as might be received in real-life clinical work. For example, including information regarding the client’s social and occupational functioning, as well as any existing health-related challenges, may have helped to further inform diagnosis and treatment recommendations made by participants. Future research may include a comprehensive biopsychosocial report of a hypothetical client that would provide participants with more detailed information of the client’s history, current functioning, and presenting problem. The vignette did not explicitly specify the treatment setting or type of treatment. Furthermore, although our measure of number of recommended treatment sessions was used in previous research (Agell and Rothblum, 1991), it limited participants to selecting a number of sessions ranging from 0 to 24. This is not consistent with many current treatments for anorexia nervosa, which may offer up to 40 sessions (Murphy et al., 2010). It is recommended that future studies offer participants the option of filling in a recommended number of sessions, or expanding the number of possible sessions that can be recommended. The use of a standardized client may provide participants with important face-to-face intake information, which would better allow for measurement of participant behaviors and non-verbal responses to the client. Future studies may seek to examine the impact of the therapeutic relationship, including rapport and empathy, on outcomes and attitudes associated with clients who present with eating disorders. Finally, this study tested the effect of a 30-year-old, heterosexual, female client’s BMI on mental health trainees’ perceptions. Further research is needed to explore the impact of other client factors on perceptions of eating disorder symptoms, such as client gender. For example, future studies may wish to explore if similar weight-related biases extend to male clients with eating disorder symptoms. Future research should also examine clinical perceptions of clients at higher weights than an “overweight” BMI.

It is strongly recommended that more educational and training initiatives feature topics like weight stigma, including increased opportunities for continuing education programs. Undergraduate and graduate college programs, particularly those in the mental health and medical fields, can include lectures on the prevalence and impact of weight stigma and the complexity of body weight (Persky and Eccleston, 2011; Brochu et al., 2018). These programs may also incorporate the use of clinical vignettes featuring clients presenting with atypical eating disorder symptoms to better prepare trainees for future practice. Additional suggested classroom interventions include those implemented by Watkins and Hugmeyer (2012), such as review of online resources promoting body size acceptance, information about weight, health, and the connection between eating disorders and high body-weight, and positive depictions of higher body-weight people. Educational initiatives may generate increased empathy and compassion toward people of a higher body-weight, and enhance perspective taking by allowing providers to consider the history and daily experiences of their clients (Gloor and Puhl, 2016).

Some mental health professionals advocate for avoidance of BMI as a marker of eating disorder diagnosis or recovery, focusing instead on psychological and physical health outcomes and behaviors (Lee and Pausé, 2016). Some of these markers may include client behaviors (e.g., dieting, laxative use, restrictive eating behaviors, excessive weighing), mood, and general functioning and engagement (e.g., occupational, social, spiritual; O’Reilly and Sixsmith, 2012). By doing so, providers may reduce weight bias and improve the early detection of eating disorders, preventing the development of more persistent and severe medical and mental health challenges.

## Author Contributions

LV designed the research study, collected the data, analyzed the data, and wrote the manuscript. JS assisted with the data collection, and construction and revision of the manuscript. PB helped to design the research study, collect the data, analyze the data, and revise the manuscript. All authors read and approved the submitted version.

## Conflict of Interest Statement

The authors declare that the research was conducted in the absence of any commercial or financial relationships that could be construed as a potential conflict of interest. The reviewer RP declared a past co-authorship with one of the authors PB to the handling Editor.
