# Supplementary material for: What’s Weight Got to Do With It? Mental Health Trainees’ Perceptions of a Client With Anorexia Nervosa Symptoms
Source: Front Psychol. 2018 Dec 17;9:2574. doi: 10.3389/fpsyg.2018.02574 (PMC6304369; doi:10.3389/fpsyg.2018.02574)
Supplement: Supplementary file 1 [file Table_1.DOCX]

Supplementary Material

What’s Weight Got to Do with It? Mental Health Trainees’ Perceptions of a Patient with Anorexia Nervosa Symptoms

Laurie A.S. Veillette, Jose Martinez Serrano, Paula M. Brochu, Ph.D.*

*** Correspondence:** Paula M. Brochu: pbrochu@nova.edu

**1 Treatment Attitudes**

(modified from Puhl, Latner, King, & Luedicke, 2013; Puhl, Luedicke, & Grilo, 2013)

Please rate your expectations of Susan’s treatment on a scale from 1 (*strongly disagree*) to 7 (*strongly agree*).

1. Susan would be frustrating to work with. R

2. Susan would be difficult to deal with. R

3. I would treat Susan with compassion and respect.

4. I would dislike treating Susan. R

5. I feel confident that I would provide quality care to Susan.

6. I feel professionally prepared to effectively treat Susan.

7. Susan would be non-compliant with my treatment recommendations. R

8. I feel that Susan would lack motivation to make lifestyle changes. R

9. Treating Susan would be professionally rewarding.

*Note*. R indicates a reverse-scored item.

# 2 Vignette

Susan Marie Greenfield is a 30-year-old, divorced mother of one. She was referred for treatment by her primary care physician after reporting a loss of appetite and a fear of weight gain. At her last physical, Susan’s height was reported to be 5’6” and she weighed 103 [132] {183} pounds. Her body mass index (BMI) is 16.6 [21.3] {29.5}, classifying her as underweight [normal weight] {overweight}. After it was determined that her loss of appetite was not related to a medical condition, Susan was referred for psychological assessment and subsequent treatment. Susan currently lives in her home in Miami, with her youngest daughter, Terry, a 13-year-old high school freshman.

Susan presented to her initial appointment on time, dressed in business casual attire that was notably too large for her frame. Susan stated that she had come from work at a bank in Miami. When asked what brought her in to the session, Susan shared that she had struggled with her weight and body image since she was a teenager. Susan reported that over the last six months she had increasingly restricted her diet, keeping a food journal to monitor caloric intake. She reported daily “weigh-ins” and weekly measurements, during which Susan measures her waist, buttocks, arms, and legs. These are also logged in her journal. Susan reported that she was “terrified” of gaining weight, stating that she did not want to develop the body shape and weight of her mother, whom Susan described as “fat” and “sloppy”. Susan stated that she would “rather die than get fat and ugly.”

Susan was asked to describe her typical day. In the morning, Susan reported that she will immediately weigh herself and journal it. She typically has a cup of coffee and half of a grapefruit for breakfast. If she requires a snack, she may consume one fat-free, plain yogurt cup. For lunch, Susan reported that she will eat half a cup of chopped cucumber, a pear, and a cup of baby carrots. In the evening, Susan will usually eat half of a baked chicken breast with a cup of steamed broccoli. Before bed, Susan will weigh herself again and journal her findings. Susan strives to maintain a caloric intake of no more than 800 calories. If she surpasses her goal, Susan shared that she will become angry with herself and may further restrict her caloric intake the following day to compensate.

When asked about her work life, Susan stated that her work life has not suffered as a result of her weight concerns and behaviors. She enjoys her position at the bank and has many positive relationships with colleagues. She stated that she enjoys baking cupcakes or cookies for the other employees at the bank, proudly boasting that she has earned a reputation for her peanut butter cookie recipe.

In her social and family life, Susan stated that her friends and family have begun making comments about her eating habits and that their concern is growing. Susan’s daughter does not like that her mother will prepare different meals for her and complains when her mother takes too long in the morning weighing and measuring herself.

When asked about her functioning before these symptoms began, Susan shared that she had always enjoyed food, often baking and cooking for friends and family, but struggled with her weight. She reported that she had been bullied in high school, describing a rather humiliating experience in a girls’ locker room during her freshman year. Susan revealed that a senior girl made several disparaging comments about Susan’s “rolls” and “cellulite.” Susan began journaling her caloric intake and weight shortly after this incident.

When asked about her relationship history, Susan revealed that she divorced her husband seven months ago, after a series of infidelities on his part. She shared that they have a “hostile” relationship and that they communicate solely through email. Susan has sole custody of their daughter, Terry, who spends summers with her father.

Susan disclosed that, as a sophomore in high school, she “thought about death maybe once or twice” but never made a plan or had any serious intent. Susan denied present suicidal and homicidal ideations. She denied any history of hallucinations or delusions. Her mental status examination revealed that Susan is oriented to place and time, her memory, judgment, and concentration are within normal ranges, and her overall affect and speech are within normal ranges. She does not evidence any symptoms of mania and denied experiencing manic symptoms in the past.
